# Communication skills of general practitioners in Nairobi, Kenya: a descriptive observational study

**DOI:** 10.3399/BJGPO.2021.0235

**Published:** 2022-06-29

**Authors:** Gulnaz Mohamoud, Robert Mash

**Affiliations:** 1 Division of Family Medicine and Primary Care, Stellenbosch University, Stellenbosch, South Africa; 2 Department of Family Medicine, Aga Khan University, Nairobi, Kenya

**Keywords:** general practice, general practitioners, primary health care, communication, consultations, private sector, primary care

## Abstract

**Background:**

High-quality primary care needs to be person-centred, and GPs must communicate effectively to ensure continuity and coordination of care. In Kenya, there is little knowledge about the quality of communication in consultations by GPs.

**Aim:**

To evaluate the quality of communication in consultations by GPs.

**Design & setting:**

Descriptive, observational study of 23 GP consultations in 13 private sector primary care facilities in Nairobi, Kenya.

**Method:**

One consultation with a randomly selected adult patient was recorded per GP, and 16 communication skills evaluated with the Stellenbosch University Observation Tool (SUOT). A total percentage score was calculated per consultation, and compared with the GPs’ demographics and the consultation complexity and duration using the Statistical Package for Social Sciences (SPSS, version 25).

**Results:**

The GPs’ median age was 30.0 years (interquartile range [IQR] 29.0–32.0) and median consultation time was 7.0 minutes (IQR 3.0–9.0). Median overall score was 64.3% (IQR 48.4–75.7). GPs demonstrated skills in gathering information, making and explaining the diagnosis, and suggesting appropriate management. GPs did not make an appropriate introduction, explore the context or patients‘ perspectives, allow shared decision making, or provide adequate safety netting. There was a positive correlation between the scores and duration of the consultations (*r* = 0.680; *P* = 0.001). The score was higher in consultations of moderate complexity (78.1, IQR 57.1–86.7) versus low complexity (52.2, IQR 45.1–66.6) (*P* = 0.012).

**Conclusion:**

Consultations were brief and biomedical by young GPs. GPs need further training in communication skills, particularly with regard to delivering person-centred consultations. Deploying family physicians to the primary care setting would also improve the overall quality of service delivery.

## How this fits in

High-quality primary care is characterised by person-centredness and effective communication skills. In the African context, patients expect person-centred consultations, but little is known about the quality of communication in consultations. This study showed that communication in private sector primary care clinics in Nairobi, Kenya is brief, biomedical, and offered by young and inexperienced GPs, who dealt with low-to-moderate complexity and mostly acute problems. Additional training and expertise are needed to deliver high-quality primary care.

## Introduction

General practice has been described as an *‘approach to delivery of health care which is characterised by whole-person medicine‘*.^1^ A biopsychosocial approach is fundamental to whole-person medicine throughout the life course and across the burden of disease.^1^ In addition, this approach takes into account the family and community context, and supports continuous and coordinated care through a collaborative relationship.^1,2^ Whole-person medicine requires primary care providers to offer person-centred care (PCC) and to have effective communication skills.^3^

In South Africa, PCC has been described as involving processes of *‘facilitation, clinical reasoning, and collaboration‘*.^3^ The facilitation process gives attention to the patient’s perspective, which may include their experience of illness, beliefs, concerns, expectations, preferences, and choices.^3^ Clinical reasoning is required throughout the consultation, and integrates the clinician’s expertise with the patient’s perspective.^3^ The collaboration process implies power-sharing and finding common ground to make mutually acceptable decisions.

Communication skills are needed to develop an effective relationship and provide structure throughout the consultation.^4^ There are specific communication skills related to each phase of the consultation: initiation, gathering information, explaining and planning, and closure.^4^ Effective communication results in better adherence to the management plan, increased patients’ and clinicians’ satisfaction, as well as reduced litigation.^5–7^ Effective communication has a direct impact on clinical outcomes and the ability of patients to self-manage chronic conditions.^8^

In Africa, there is little evaluation of effective communication in the primary care consultation. A study from the public sector in Uganda found that patients expected PCC, although it was not measured.^9^ A South African study in the public sector found significant gaps in the ability of primary care providers to give PCC.^10^ Primary care delivery in the Kenyan private sector relies on GPs;^11^ however, GPs without postgraduate training may not have been trained in the communication skills that are essential to the practice of PCC.^12,13^ Some family physicians have completed postgraduate training with an emphasis on effective communication, but numbers are very small despite the availability of five training programmes in Kenya.^14^ Not much is known about the quality of communication in consultations offered by GPs. Hence, the aim of this study was to evaluate the quality of GPs’ communication in consultations as an essential component of high-quality primary care in private sector settings in Nairobi, Kenya.

## Method

### Study design and setting

This was a descriptive observational cross-sectional study. This study was conducted in 13 primary care clinics attached to a tertiary hospital within Nairobi, operated by one private healthcare organisation. These clinics offered promotive, preventive, and treatment services to all age groups across Nairobi. Each clinic had a pharmacy and laboratory, and could refer patients to specialist clinics (including family medicine) at the tertiary hospital. An electronic health record allowed clinicians to access patients’ information at all of these facilities. There were 25 GPs working in these facilities. Most of the patients attending these clinics had health insurance. These private sector clinics had no empanelment of practice populations and were not obligated to provide gatekeeping for the hospital. This model of care, within a private healthcare organisation, was very different to the model of primary health care in the public sector.

In Kenya, GPs usually have no postgraduate education in family medicine. It is only recently that a 4-year Masters of Medicine degree was introduced that leads to registration as a specialist in family medicine. The number of graduates is very small and family physicians may work at district hospitals as well as primary care.

Primary care is undergoing a transition in Kenya as chronic non-communicable diseases, such as diabetes, ischaemic heart disease, and stroke, increase in importance alongside HIV, tuberculosis, malaria, and other acute infectious diseases.^15^ Among the urban populations served by this private sector organisation, the relative contribution of non-communicable chronic conditions is likely to be higher than the national average.

#### Study population

All 25 GPs were invited to contribute one audiorecording of a consultation. The intention was to describe the overall collective quality of consultations and not that of individual GPs. The reliability of 25 observations of these GPs, when regarded as a collective or single entity, was thought to be aligned with workplace assessment methods.^16^ This number of observations was feasible to assess and a similar approach was used in a previous study in South Africa.^10^

### Data collection

One adult patient was selected by the lead author using computer-generated random numbers (Microsoft Excel) from the GP’s patient list for that day. After obtaining consent from both the patient and the GP, the consultation was recorded using a discrete microrecorder. Consultations were routinely conducted in English. Basic demographic data related to each GP was also collected.

The audiorecordings were assessed using the SUOT. The tool was developed from the evidence-based Calgary–Cambridge guide to consultation skills.^4^ The tool was previously used to research primary care consultations in South Africa.^10^ This tool is also used in postgraduate family medicine assessment in South Africa and Kenya, and is published in the *South African Family Practice Manual*.^17,18^ The SUOT was piloted to assess the reliability of the assessor and feasibility in the study setting.

The SUOT evaluates 16 different consultation skills as ’not done‘ (score = 0), ‘partially done‘ (score = 1), ‘fully done‘ (score = 2), or ‘not applicable‘ for the specific consultation.

### Data analysis

All data were entered into a Microsoft Excel spreadsheet and checked for errors or omissions. Data were analysed using the SPSS (version 25).

The principal researcher was trained on the SUOT by the second author and inter-rater reliability was confirmed using four randomly selected recordings. The SUOT has been shown to have good inter-rater and intra-rater reliability in South Africa.^10^ A good level of agreement was indicated by a Κ of 0.875 (95% confidence interval [CI] = 0.284 to 0.999). High intra-rater reliability was shown with an intra-class correlation coefficient of 0.98 (95% CI = 0.814 to 0.999).

Reasons for the encounter and the diagnoses made in each consultation were coded using the International Classification of Primary Care, 2nd edition (ICPC-2).^19^ Consultations were categorised into different complexities based on the number of reasons for the encounter and diagnoses. Low complexity was defined as one or two reasons for the encounter or one diagnosis, moderate complexity as three or four reasons for the encounters or two diagnoses, and high complexity as five or more reasons for the encounter or three or more diagnoses.^20^ The Practical Approach to Care Kit (PACK) guideline was used to assess the appropriateness of the management plan. The PACK is an integrated and evidence-based guideline used in adult primary care for the management of common symptoms and chronic conditions.^21^

The frequencies and percentages for the different evaluation categories for individual communication skills were analysed as well as the total consultation scores as a percentage out of 32. The relationships between the duration of the consultation, age of the GP and years of experience, and the total consultation score were analysed using Spearman’s correlation. The relationships between the complexity of the consultation and sex of the GPs with the total consultation score were analysed using the Mann-Whitney *U* test. This test was also used to investigate the relationship between the duration of consultations and the complexity of the cases.

## Results

The response rate of the GPs was 92.0% (*n* = 23/25), with nine males and 14 females. The GPs had a median age of 30.0 years (IQR 29.0–32.0) and a median of 3 years of experience after graduation (IQR 3.0–6.0). The consultations were of low (*n* = 12, 52.2%) and moderate (*n* = 11, 47.8%) complexity. The median consultation time was 7.0 minutes (IQR 3.0–9.0).

Table 1 shows the main reasons for encounter and diagnoses. Most symptoms and diagnoses were categorised into gastrointestinal, respiratory, musculoskeletal, and general domains.

**Table 1.** Main reasons for encounter and diagnoses in the consultations

Number	ICPC domains for reasons for encounter	*n* (%), *n* = **49**	Number	ICPC domains for diagnoses	*n* (%),*n* = **31**
1	Gastrointestinal	13 (26.5)	1	Gastrointestinal	10 (32.3)
2	Respiratory	9 (18.4)	2	Musculoskeletal	7 (22.6)
3	Musculoskeletal	6 (12.2)	3	Respiratory	6 (19.4)
4	General	6 (12.2)	4	Female genital	4 (12.9)
5	Neurological	5 (10.2)	5	Skin	1 (3.2)
6	Female genital	5 (10.2)	6	Cardiovascular	1 (3.2)
7	Urological	2 (4.1)	7	Male genital	1 (3.2)
8	Skin	1 (2.0)	8	Urological	1 (3.2)
9	Male genital	1 (2.0)			
10	Eye	1 (2.0)			

ICPC = International Classification of Primary Care.

**Figure 1.**
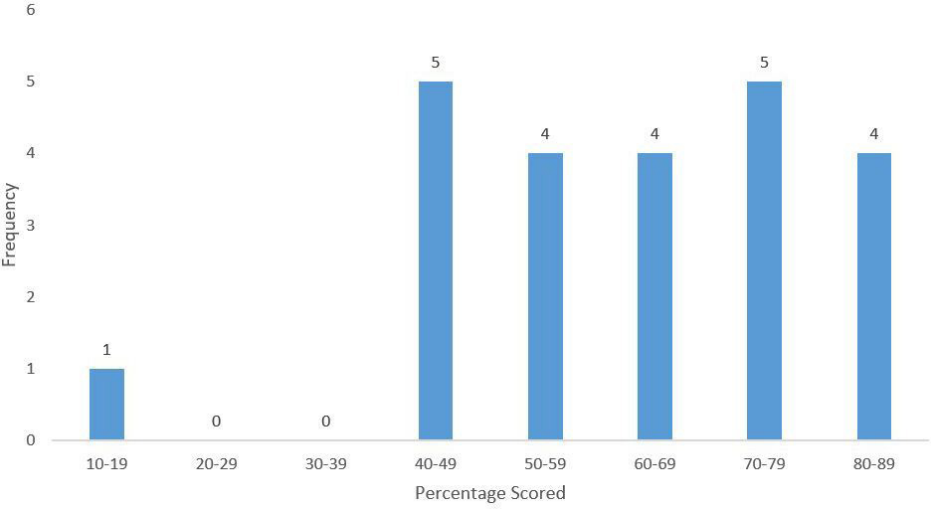
Distribution of total percentage scores for the consultations (*n* = 23)

Figure 1 shows the distribution of total percentage scores, with a median score of 64.3% (IQR 48.4–75.7). Table 2 indicates performance for each consultation skill. In >50% of consultations, the GPs did not make an appropriate introduction or greeting, nor did they explore the family and social context, or relate their explanation to the patient’s perspective. Similarly, in >50% of the consultations, they only partly succeeded in confirming the patient’s problem list, encouraged the patient to tell their story, and understood the patient’s perspective.

**Table 2.** Evaluation of consultation skills

Number	Consultation skill^a^	Not done,*n* (%)	Partially done,*n* (%)	Fully done,*n* (%)
1	Makes appropriate greeting or introduction, and demonstrates interest and respect (*n* = 23)	12 (52.2)	5 (21.7)	6 (26.1)
2	Identifies and confirms the patient’s problem list or issues (*n* = 23)	9 (39.1)	12 (52.2)	2 (8.7)
3	Encourages patient’s contribution or story (*n* = 23)	1 (4.3)	12 (52.2)	10 (43.5)
4	Makes an attempt to understand the patient’s perspective (*n* = 23)	9 (39.1)	13 (56.5)	1 (4.3)
5	Thinks family, and obtains relevant family, social, and occupational information (*n* = 23)	12 (52.2)	8 (34.8)	3 (13.0)
6	Obtains sufficient information to ensure no serious condition is likely to be missed (*n* = 23)	1 (4.3)	3 (13.1)	19 (82.6)
7	Appears to make a clinically appropriate working diagnosis (*n* = 23)	1 (4.3)	2 (8.7)	20 (87.0)
8	There is a clear explanation of the diagnosis and management plan (*n* = 18)	0 (0.0)	2 (11.1)	16 (88.9)
9	Gives patient an opportunity to ask for other information and/or seeks to confirm patient’s understanding (*n* = 18)	2 (11.2)	8 (44.4)	8 (44.4)
10	The explanation takes account of and relates to the patient’s perspective (*n* = 20)	13 (65.0)	3 (15.0)	4 (20.0)
11	Involves the patient where appropriate in decision making (*n* = 23)	3 (13.0)	7 (30.4)	13 (56.5)
12	Chooses an appropriate management plan (*n* = 20)	0 (0.0)	1 (5.0)	19 (95.0)
13	Shows a commitment to coordination of care (*n* = 15)	0 (0.0)	1 (6.7)	14 (93.3)
14	Shows a commitment to continuity of care (*n* = 20)	6 (30.0)	1 (5.0)	13 (65.0)
15	Closes consultation successfully (*n* = 21)	5 (23.8)	5 (23.8)	11 (52.4)
16	Provides appropriate safety netting for the patient (*n* = 19)	7 (36.8)	5 (26.4)	7 (36.8)

^a^ *n* differs between skills, as not all skills were relevant in every consultation.

The GPs fully performed (had a score of 2) four of the 16 skills in >60% of consultations, which were as follows: obtaining sufficient information; making an appropriate diagnosis; providing a clear explanation; and formulating an appropriate management plan. However, there was less shared decision making. The GPs demonstrated a commitment to coordination and continuity of care in the majority of relevant consultations, although safety netting and closure were not fully addressed.

The median percentage score was significantly higher in consultations of moderate complexity (78.1, IQR 57.1–86.7) compared with low complexity (52.2, IQR 45.1–66.6) (*P* = 0.012). There was a significant positive correlation between an increasing consultation score and longer duration of the consultations (*r* = 0.680; *P* = 0 .001). There was no significant relationship between the age (*r* = 0.072; *P* = 0.743), experience of the GPs (*r* = −0.164; *P* = 0.454), and sex (*P* = 0.614) with the consultation score. Moderate complexity consultations took longer (median = 9.0, IQR 6.6–12.3 minutes) than low complexity consultations (median = 4.0, IQR 2.8–7.0 minutes), although the difference did not reach significance (*P* = 0.095).

## Discussion

### Summary

Primary care was offered by young GPs, who conducted brief consultations of low-to-moderate complexity. They were able to obtain sufficient biomedical information, make an appropriate diagnosis, and formulate and explain an appropriate management plan. Gaps were found in the provision of whole-person medicine and PCC, with little attention being paid to the patient’s perspective and context. In the majority of consultations, there was a commitment to coordinating care within the practice and to ongoing care. The GPs varied considerably in their provision of safety netting. The consultation score improved with increasing complexity of the problems and length of the consultation.

### Strengths and limitations

There are many tools available to evaluate consultations and PCC in particular; however, there is no international consensus on which tool is best to use or which model of the consultation is most applicable.^3,22^ The authors believe the SUOT was a reasonable choice as it was evidence-based and regarded as valid in the local context.

To adequately measure each individual, a sample size of at least 8–10 consultations per GP would have been needed. However, such a large sample of 200–250 consultations would not have been practical to collect in the context or feasible to assess with the resources available. It is possible that more precise results for the group as a whole would be obtained with a larger sample of consultations.

The presence of the audiorecorder may have influenced the GPs to perform better in their consultations (the Hawthorne effect) and scores might be lower in unobserved consultations. However, the audiorecorder was a small discreet device and would have been easy to overlook during the consultation process. Non-verbal communication could not be assessed. These findings cannot be generalised to other GPs working in the private sector. Nevertheless, it is likely that GPs with a similar level of training and working in a comparable context would perform similarly.

There were relatively young GPs in this study population and the authors’ perception was that they were intending to pursue careers in other disciplines. Perhaps the lack of incentives/remuneration and the lack of comprehensive primary care in these clinics may also have dissuaded more experienced GPs, who saw this as their career choice, from working in these clinics.

### Comparison with existing literature

The initial aspects of building rapport and showing interest are an important part of the facilitative process in PCC,^3^ and were not demonstrated by the majority of GPs. A critical component of PCC is understanding the patient’s perspective.^23^ During the facilitative and collaborative processes of the consultation little opportunity was given to the patients to voice their perspectives or express an opinion on the treatment plan. These deficiencies may be owing to a lack of relevant training at both undergraduate and postgraduate levels,^4,24^ as well as a lack of role models in the healthcare system.^24^

Poor PCC might be expected to reduce patient satisfaction;^25^ however, a study in the same setting reported that patients had high levels of satisfaction with their consultations by the same GPs.^6^ The high level of satisfaction could be owing to low expectations, as well as the low complexity of the cases that did not demand a more in-depth approach.^6,26^

GPs did not explore patients’ psychosocial and occupational history. This may be owing to the low complexity of problems, although even relatively simple or common problems may have a link to the living or working environment, and ignoring these aspects may lead to a superficial understanding of the problem.^3^

GPs were able to diagnose and make appropriate treatment plans; however, there was little shared decision making and the diagnosis was not explained in detail. Despite this, the patients appeared to accept the doctor’s advice and treatment. An overly biomedical approach was also noted in primary care providers in studies carried out in the public sectors of South Africa and Kenya.^10,27^

The increase in chronic diseases in Africa will require better continuity and coordination of care.^6,28,29^ High levels of parallel coordination were observed within the clinic, but not referrals to the hospital (sequential coordination). This may be owing to the low-to-moderate complexity of the cases and lack of patients with chronic conditions.

This study also showed some commitment to relational continuity. In contrast, studies carried out in South Africa in the public sector showed a gap in relational continuity despite patients presenting with chronic conditions.^10,30^ This difference between private and public healthcare sectors could be owing to smaller practice populations in the private sector, consistent primary care providers, use of electronic records and appointment systems, and easy access via medical insurance to ongoing care.^26^

Safety netting is a critical component of consultations in primary care, as it contributes to better diagnostic and clinical outcomes.^31^ Safety netting was not evident in the consultations, which may be owing to the low complexity of cases or a gap in the training of GPs.

In high-income settings, consultations by GPs are often 10–15 minutes,^32,33^ which contrasts with the average time of 7 minutes in the present study. This may reflect the low complexity of the cases and lack of chronic conditions, as well as lack of PCC. It is known that patients prefer longer consultations, which result in more opportunities for preventive and health promotion advice, as well as a reduction in the number of medications prescribed.^34,35^

Highly complex cases were not seen and this finding is contrary to a study in the public sector in South Africa, where GPs were expected to see cases of this complexity.^10^ Interestingly, in the present study, as the consultations became more complex the doctors became more holistic. This may point towards their ability to be more person-centred when they perceived that the patient’s problems required a more holistic approach.

The low-to-moderate complexity of problems addressed by GPs suggests that care in these primary care clinics was of limited scope and not fully comprehensive. A previous study in this setting also found that this is how patients perceived the clinics.^26^ There was no gatekeeping required for access to hospital care and in fact family medicine services were based in the tertiary hospital. Patients with more complex problems, therefore, may have referred themselves to the hospital. In most cost-effective health systems, patients with chronic conditions are routinely managed by trained GPs in primary care,^14,36^ whereas in this system these patients were most likely managed by specialists in the tertiary hospital.

### Implications for practice

In-service training programmes for these GPs should target the deficiencies in their communication skills. Key areas to focus on include initiating the consultation, eliciting and acknowledging the patient’s perspective, and gathering holistic information. Such training should involve theory, modelling, and practice with feedback. This could be embedded in the existing continuing professional development and spearheaded by the Department of Family Medicine. There could also be opportunities for a community of practice between the GPs who might review recordings of consultations with support from family physicians. Including more communication skills training in the undergraduate curriculum may be necessary and postgraduate training in family medicine could also be a valuable avenue for doctors to learn PCC.^37,38^

Focusing on the service delivery design in this setting could also address the limited scope and comprehensiveness of primary care.^6,26^ Increased involvement of the family medicine department in the primary care setting and deploying the newly graduated family physicians to these clinics could improve communication and service delivery.

In conclusion, consultations in these primary care clinics were carried out by young GPs with no postgraduate training in family medicine. Consultations were brief and had a biomedical approach for patients with acute problems of low-to-moderate complexity. Although GPs showed competency in the medical management of their patients, they lacked skills in whole-person medicine. The findings, combined with other studies in the same context, suggest that this private healthcare system is not yet offering fully comprehensive primary care. Attention should be given to the training of doctors and to the service delivery design.
